# Microscale Biosignatures and Abiotic Mineral Authigenesis in Little Hot Creek, California

**DOI:** 10.3389/fmicb.2018.00997

**Published:** 2018-05-25

**Authors:** Emily A. Kraus, Scott R. Beeler, R. Agustin Mors, James G. Floyd, GeoBiology 2016, Blake W. Stamps, Heather S. Nunn, Bradley S. Stevenson, Hope A. Johnson, Russell S. Shapiro, Sean J. Loyd, John R. Spear, Frank A. Corsetti

**Affiliations:** ^1^Geo- Environmental- Microbiology Laboratory, Department of Civil and Environmental Engineering, Colorado School of Mines, Golden, CO, United States; ^2^Department of Earth and Planetary Sciences, Washington University in St. Louis, St. Louis, MO, United States; ^3^Laboratorio de Paleobiología y Geomicrobiología Experimental, Centro de Investigaciones en Ciencias de la Tierra (CONICET-UNC), Córdoba, Argentina; ^4^Department of Microbiology and Plant Biology, The University of Oklahoma, Norman, OK, United States; ^5^Department of Earth Sciences, University of Southern California, Los Angeles, CA, United States; ^6^Department of Biological Sciences, California State University, Fullerton, Fullerton, CA, United States; ^7^Geological and Environmental Sciences, California State University, Chico, Chico, CA, United States; ^8^Department of Geological Sciences, California State University, Fullerton, Fullerton, CA, United States

**Keywords:** carbonate–silicate microbialite, hot spring biofilm, biosignature, stromatolite, microbial mat

## Abstract

Hot spring environments can create physical and chemical gradients favorable for unique microbial life. They can also include authigenic mineral precipitates that may preserve signs of biological activity on Earth and possibly other planets. The abiogenic or biogenic origins of such precipitates can be difficult to discern, therefore a better understanding of mineral formation processes is critical for the accurate interpretation of biosignatures from hot springs. Little Hot Creek (LHC) is a hot spring complex located in the Long Valley Caldera, California, that contains mineral precipitates composed of a carbonate base (largely submerged) topped by amorphous silica (largely emergent). The precipitates occur in close association with microbial mats and biofilms. Geological, geochemical, and microbiological data are consistent with mineral formation via degassing and evaporation rather than direct microbial involvement. However, the microfabric of the silica portion is stromatolitic in nature (i.e., wavy and finely laminated), suggesting that abiogenic mineralization has the potential to preserve textural biosignatures. Although geochemical and petrographic evidence suggests the calcite base was precipitated via abiogenic processes, endolithic microbial communities modified the structure of the calcite crystals, producing a textural biosignature. Our results reveal that even when mineral precipitation is largely abiogenic, the potential to preserve biosignatures in hot spring settings is high. The features found in the LHC structures may provide insight into the biogenicity of ancient Earth and extraterrestrial rocks.

## Introduction

Differentiating morphological biosignatures from abiogenic mineral assemblages remains a problem in the interpretation of the evolution of life in the geologic rock record and for the search for life on other planets. The environments of early Earth and Mars are thought to have been similar, containing aqueous geothermal activity, which could provide the potential to preserve traces of life (McKay and Stoker, 1989; Walter and Des Marais, 1993; Preston et al., 2008; Wordsworth, 2016). Detection of surface silica deposits associated with hydrothermal features on Mars (Squyres et al., 2008) and their resemblance to biotically-influenced silica structures on Earth (Ruff and Farmer, 2016) highlights the need for further study of active modern hot springs with silica precipitation. The discovery of hot-spring-associated biosignatures on Mars has been a goal of astrobiological research for decades, largely guided by insight gained from Earth analog systems (NASA, 1995; Cady et al., 2003).

Earth’s modern hot springs exhibit strong thermal and chemical gradients that generate energetically favorable redox conditions for microbial life (Meyer-Dombard et al., 2005, 2011; Spear et al., 2005; Inskeep et al., 2010). Rapid mineral precipitation tends to occur in these systems as inorganic carbon- and silica-containing waters reach the surface and interact with the atmosphere. When these waters reach the surface, rapid physico-chemical changes occur due to degassing, cooling, evaporation, and water mixing, which can drive carbonate and silica precipitation abiotically (Fouke et al., 2000; Konhauser et al., 2004; Pentecost, 2005; Fouke, 2011). Due to rapid mineralization, microorganisms and/or traces of their activity can be preserved in the rock record (e.g., Cady and Farmer, 1996). Recent findings have demonstrated the ability of similar silica-rich deposits to preserve some of the earliest signs of life on Earth (Djokic et al., 2017), but abiotically-synthesized microstructures resembling biological morphologies have been shown to self-assemble in an experimental setting, casting doubt on solely using morphological features as biogenic indicators (García-Ruiz et al., 2003).

Microorganisms can induce mineral precipitation by changing local chemical microenvironments on micrometer scales through metabolic activity. For example, metabolic reactions such as photosynthesis, sulfate reduction, and denitrification have been shown to drive carbonate mineral precipitation by increasing relative alkalinity, thus increasing the saturation state of calcite (Dupraz and Visscher, 2005; Visscher and Stolz, 2005; Baumgartner et al., 2006; Chrachri et al., 2018). In addition, microbes can control mineral nucleation by the production of negatively charged surfaces, like exopolymeric substances (EPS), providing a surface for mineral nucleation, which has been observed in silica precipitation in hot springs (Konhauser and Ferris, 1996; Farmer and Des Marais, 1999; Phoenix et al., 2000; Benning et al., 2004). Thus, several processes can influence carbonate precipitation and silicification in different ways. Microbial control can be direct, as in carbonates (Dupraz and Visscher, 2005), or marginal to absent, as in silicification, by imparting an influence on the resulting mineral textures (Konhauser et al., 2004).

Disentangling the main physio-chemical and microbiological controls in mineral precipitation is necessary to better understand biosignature preservation in the sedimentary record; thus, the study of modern active hot spring systems yields valuable insight into Earth’s history and beyond. Little Hot Creek (LHC) is a hot spring system in the Long Valley Caldera of California, containing mineral precipitates with a carbonate base and an amorphous silica/carbonate top. The precipitates occur in close association with microbial mats and biofilms in the spring. The phage community structure, geochemistry, isotopic characteristics, mineralogy, microbial community of LHC sediments, and microbial mat structures have been described in some detail previously (Breitbart et al., 2004; Vick et al., 2010; Bradley et al., 2017; Wilmeth et al., in review). The mineral precipitate microbial communities, physical structure, and mineralogy have not been characterized, affording an opportunity to investigate the generation of silica- and carbonate-based biosignatures. Here, we investigate the biogenicity of LHC precipitates using geochemical modeling, petrography, microscopy, high-throughput DNA sequencing, and isotopic data to better inform how the structures formed.

## Materials and Methods

### Site Description and Aqueous Geochemistry

**FIGURE 1:**
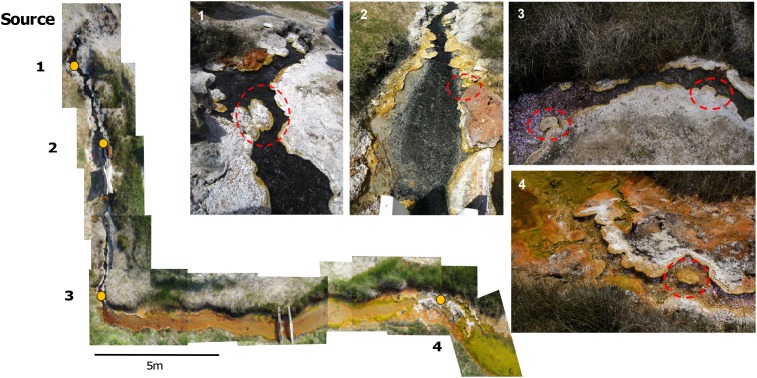
A photomosaic of Little Hot Creek 1. Sampling sites are indicated by yellow dots. Dashed red circles show the precipitate structures that were removed from LHC for sampling.

Little Hot Creek 1 (LHC) (Vick et al., 2010) is a 28-m-long channelized hot spring in the hydrothermally-active Long Valley Caldera of California at 37°41′26.1^′′^N 118°50′39.9^′′^W. Samples for water geochemistry and four mineralized structures within the hot spring were collected for biological, isotopic, and petrographic sampling under a United States Forest Service Research Permit (#MLD15053) (**Figure 1**). All structures were rooted in the spring bottom or side and ranged in size, with the largest structure approximately 15 cm long, 10 cm wide, and 9 cm thick. The above-water mineral surfaces were covered in biofilms of varying colors and/or white grainy material. Spring water was collected several centimeters directly upstream of the chosen mineralized structures via syringe and passed through a 0.45 μm filter to fill 15 ml Falcon^®^ tubes with no headspace for triplicate measurements of anions and cations. Samples for cation analysis were acidified with drops of nitric acid and triplicate samples of four milliliters of 0.45 μm filtered water was injected into pre-evacuated Exetainer^®^ vials (Labco Limited, Lampeter, Wales, United Kingdom) for dissolved inorganic carbon (DIC) measurements. Temperature and pH were measured at the point of water collection with a Mettler Toledo SevenGo Duo^TM^ pH meter (Mettler Toledo, Columbus, OH, United States) at each site.

To empirically assess the isotopic evolution of dissolved inorganic carbon downstream at LHC, one source fluid degassing experiment was performed on-site contemporaneously with sample collection. This experiment was conducted on spring source waters to remove potential impacts from spring channel biology and mineral precipitates. One and a half liters of water was collected from the source of LHC in an open container and placed on a stir plate to mimic fluid turbulence exhibited along the flow path (the complexity of LHC outflow morphology produces local variability in turbulence; stirring was meant to mimic but not perfectly reconstruct turbulence). At 30-s intervals 4 ml of water was removed, passed through a sterile 0.45 μm filter, and injected into an evacuated Exetainer^®^ vial to analyze for DIC concentration and isotopic composition. Temperature and pH were recorded concurrently at each sampling time point. The entire duration of the source fluid experiment lasted 15 min.

Cation and anion concentrations were analyzed using inductively coupled plasma atomic emission spectroscopy (ICP-AES; Optima 5300, PerkinElmer, Fremont, CA, United States) and ion chromatography (IC; ICS-90, Dionex, Sunnyvale, CA, United States), respectively. The maximum allowable sodium concentration in the ICP-AES instrument is 500 mg/L and the maximum allowable chloride concentration in the IC instrument is 300 mg/L; therefore, samples were diluted accordingly using ultrapure water and then acidified to a pH of less than 2 using nitric acid for ICP only. Dissolved inorganic carbon (DIC) concentrations and δ^13^C values were determined on waters from LHC and the degassing experiment using a cavity ringdown spectrometer [(CRDS), 2121-i, Picarro, Santa Clara, CA, United States]. Samples were acidified using an AutoMate carbonate preparation device (AutoMate FX, Inc., Bushnell, FL, United States) to quantitatively convert DIC to CO_2_(g). CO_2_(g) is passed via ultra-high purity N_2_ carrier gas to the CRDS. The CRDS provides simultaneous DIC concentration and δ^13^C data. DIC concentration and δ^13^C replicates are generally better than ±0.3 mM and 0.5‰, respectively.

Calculation of saturation indices and geochemical modeling were performed with PHREEQC-I version 3.3.7 (Parkhurst and Appelo, 2013). PHREEQC-I enables modeling of saturation state with respect to temperature and elemental activities. This allows for the ability to assess changes in saturation state as these parameters change during physico-chemical processes such as evaporation, cooling, and degassing. Measured source water values were used as the initial parameters for all geochemical modeling performed.

### Solid Carbonate Stable Isotope Analysis

Solid samples were powdered using either a rotary tool (Dremel Co., Racine, WI, United States) fitted with a 1.5 mm carbide drill bit or mortar and pestle. Powdered samples (5–7 mg) were placed into evacuated Exetainer^®^ vials in triplicate. Carbonate powders were then dissolved in 3 ml of 10% phosphoric acid overnight. Acid-produced CO_2_(g) was passed via ultra-high purity N_2_ carrier gas to the CRDS. The CRDS provides simultaneous total inorganic carbon (TIC) content and δ^13^C data. TIC concentration and δ^13^C replicates are generally better than ±0.2 wt% and 0.1‰, respectively.

### Petrography and Microscopy of LHC Precipitates

Large (51 mm × 75 mm) and small (27 mm × 46 mm) format thin sections were made from several of the samples. Photomosaics of select thin sections were produced on a Zeiss Axioscope petrographic microscope (Carl Zeiss Microscopy, LLC, Thornwood, NY, United States) in the petrography lab at the University of Southern California. Samples of LHC mineral precipitates and associated biofilms were processed for biologic microscopy. Partially lithified precipitate material was homogenized by finely grinding approximately 1 g of material with a mortar and pestle with sterile phosphate buffered saline. The homogenized material was placed onto glass slides and imaged on an Olympus BX41 (Olympus Corporation, Shinjuku, Tokyo, Japan). Images were collected under 400× using brightfield, fluorescent green light (540 – 560 nm) to detect the presence of photosynthetic organisms, and DAPI (4′6′-diamidino-2-phenylindole) stain under UV light to highlight DNA in organisms associated with mineral precipitates. Environmental scanning electron microscopy (ESEM) was done on mineral precipitates from Sites 3 and 4 (**Figure 1**). Small sections from the top mineral precipitate (approximately 3 mm × 3 mm × 3 mm) were prepared without chemical fixation, sputter coating, or dehydration steps. ESEM was performed on a Hitachi TM-1000 Tabletop SEM (Hitachi Global, Tokyo, Japan) equipped with a back-scatter electron (BSE) detector, an accelerating voltage of 15 kV, and a working distance of 8.4 – 8.7 mm.

### DNA Extraction and Sequencing From LHC Precipitates

Sterile spatulas were used to scrape partially lithified, carbonaceous material and overlying green/brown biofilm from the upstream, submerged edge of each of the four mineralized structures. Three technical replicates were collected from each. Three technical replicate samples were also taken from the above-water, precipitate-top portions of the structures at Sites 1, 2, and 4 (**Figure 1**). Three samples for metagenomic sequencing were collected from the leading, submerged edge of the mineral structure at Site 4. Samples were immediately suspended in 750 μL Xpedition^TM^ Lysis/Stabilization Solution (Zymo Research Co., Irvine, CA, United States), and homogenized for 1 min on-site using a custom designed lysis head attached to a reciprocating saw. Samples in the field were then maintained at room temperature (∼25°) stably in the Lysis/Stabilization Solution for several hours and stored at -20°C in the laboratory until completion of nucleic acid extraction. Within 2–3 days, DNA was extracted from these preserved samples using the Zymo Research Xpedition Soil/Fecal DNA MiniPrep Extraction kit (Zymo Research Co.) following manufacturer’s instructions.

Libraries of partial bacterial, archaeal, and eukaryotic small subunit (SSU) rRNA genes were amplified from each DNA extraction using PCR with primers that spanned the V4 and V5 hypervariable regions of the 16S ribosomal RNA gene (16S rRNA gene) between position 515 and 926 (*Escherichia coli* numbering), producing a ∼400 bp fragment for Bacteria and Archaea, and a 600 bp fragment for the Eukarya 18S rRNA gene. These primers evenly amplify a broad distribution of SSU rRNA genes from all three domains of life (Parada et al., 2015). The forward primer 515F-Y (**GTA AAA CGA CGG CCA G C**CG TGY CAG CMG CCG CGG TAA-3′) contains the M13 forward primer (in bold) fused to the gene-specific forward primer (underlined), while the reverse primer 926R (5′-CCG YCA ATT YMT TTR AGT TT-3^′′^) was unmodified from Parada et al. (2015). 5 PRIME HotMasterMix (Quanta Biosciences, Beverly, MA, United States) was used for all reactions at a final reaction volume of 50 μL. Reactions were purified using Agencourt^®^ Ampure^®^ XP paramagnetic beads (Beckman Coulter Inc., Indianapolis, IN, United States) at an 0.8x final concentration. After purification, 4 μL of PCR product was used in a barcoding reaction to attach a unique 12 bp barcode to each library in duplicate 50 μL reactions. Duplicate reactions were pooled, purified using AmpureXP beads to a final volume of 40 μL, quantified using the Qubit^TM^ dsDNA HS assay kit (Thermo Fisher Scientific Inc., Waltham, MA, United States), and pooled in equimolar amounts before concentration using an Amicon^®^ Ultra 0.5 ml centrifugal filter unit with Ultracel-30K membrane (Millipore Sigma, Billerica, MA, United States) to a final volume of 80 μL. To mitigate the effects of reagent contamination (Salter et al., 2014), triplicate extraction blanks (DNA extraction with no sample addition) and negative PCR controls (PCR with no template DNA added) were sequenced. The pooled, prepared library was then submitted for sequencing on the Illumina MiSeq (Illumina Inc., San Diego, CA, United States) using V2 PE250 chemistry at the Oklahoma Medical Research Foundation (OMRF) Clinical Genomics Center^1^.

Small sub-unit (SSU) rRNA gene analyses were carried out within QIIME version 1.9.1 (Caporaso et al., 2010b). Briefly, paired-end reads were joined using PEAR (Zhang et al., 2014), barcodes were extracted, and de-multiplexed prior to operational taxonomic unit (OTU) clustering. Chimeras were filtered prior to clustering using usearch61 (Edgar, 2010) and representative sequences for each OTU were assigned a taxonomic identity by mothur (Schloss et al., 2009) against the SILVA r128 database (Yilmaz et al., 2013) clustered to a 97% similarity. Finally, sequences were aligned with PyNAST (Wang et al., 2007; Caporaso et al., 2010a) against the SILVA r128 database and FastTree (Price et al., 2010) was used to produce a phylogenetic tree to generate a weighted UniFrac distance matrix (Lozupone and Knight, 2005). A BIOM file (McDonald et al., 2012) was generated and used to generate bar charts of the relative abundances of OTUs in each sample. A phylogenetic tree of OTUs belonging to the Cyanobacteria phylum with greater than 100 sequence counts (40 OTUs total) was constructed. Evolutionary history was inferred by using the Maximum Likelihood method based on the Tamura-Nei model (Tamura and Nei, 1993). Initial tree(s) for the heuristic search were obtained automatically by applying Neighbor-Join and BioNJ algorithms to a matrix of pairwise distances estimated using the Maximum Composite Likelihood (MCL) approach, and then selecting the topology with superior log likelihood value. A total of a thousand trees were produced to generate bootstrap values. All positions containing gaps and missing data were eliminated. A total of 417 positions were in the final dataset. Evolutionary analyses were conducted in MEGA7 (Kumar et al., 2016).

The metagenomic libraries were prepared from three samples of precipitate material collected from the upstream edge of the Site 4 mineral structure using the Nextera XT library preparation kit (Illumina Inc., San Diego CA, United States) following manufacturer’s instructions. Briefly, DNA from each sample was normalized to a total amount of 1 ng as input into the tagmentation reaction. After limited-cycle amplification, samples were cleaned, normalized using a bead-based method (Illumina Inc.), pooled at an equimolar ratio, and sequenced on the Illumina NextSeq 500 using high output PE150 chemistry at the OMRF Clinical Genomics Center. Metagenomic sequencing reads were demultiplexed and had barcodes removed prior to being uploaded to MG-RAST (Meyer et al., 2008; Wilke et al., 2015). The MG-RAST pipeline removed adapters and completed a quality control step before analysis and annotated using the KEGG Orthology database (Kanehisa, 2002) for functionality and against the RefSeq database (Pruitt et al., 2007) for taxonomic assignments.

## Results

### Little Hot Creek Water Geochemistry

The water chemistry of Little Hot Creek exhibits substantial variability from source to outflow (**Table 1**). Waters near the hot spring source are hot and mildly acidic (81.2°C, and pH = 6.7 at the sampling location closest to source). Waters become cooler and more alkaline as they move downstream over a 28 m transect (70.7°C, and pH = 7.45 at the final sampling location). The concentration of DIC decreases from 15.7 mM closest to the source to 12.8 mM at the output. All other measured geochemical parameters display little variability along the flow path (**Table 1**). Data collected in 2015 from LHC show a decrease in the dissolved silica concentration from 1.49 mM near the source to 1.36 mM at the output (2015 Agouron International Geobiology Course, unpublished data). LHC waters are undersaturated with respect to amorphous silica at all sampling locations.

**Table 1:** Water chemistry values along the flow path of Little Hot Creek.

Site	pH	T (°C)	Ω_Calcite_	DIC (mM)	Na^+^ (mM)	K^+^ (mM)	Ca^2+^ (mM)	F^-^(mM)	Cl^-^(mM)	SO_4_^2-^ (mM)
1	6.70	81.2	2.1	15.7	16.8	0.7	1.3	0.5	5.0	1.1
2	6.80	79.6	1.5	14.9	17.5	0.7	0.7	0.5	5.3	1.2
3	7.07	76.7	2.1	13.7	17.5	0.7	0.7	0.5	4.9	1.1
4	7.45	70.7	7.2	12.8	17.3	0.8	1.0	0.5	5.1	1.1

*Site 1 is closest to the hot spring source and Site 4 is closest to the output.*

**FIGURE 2:**
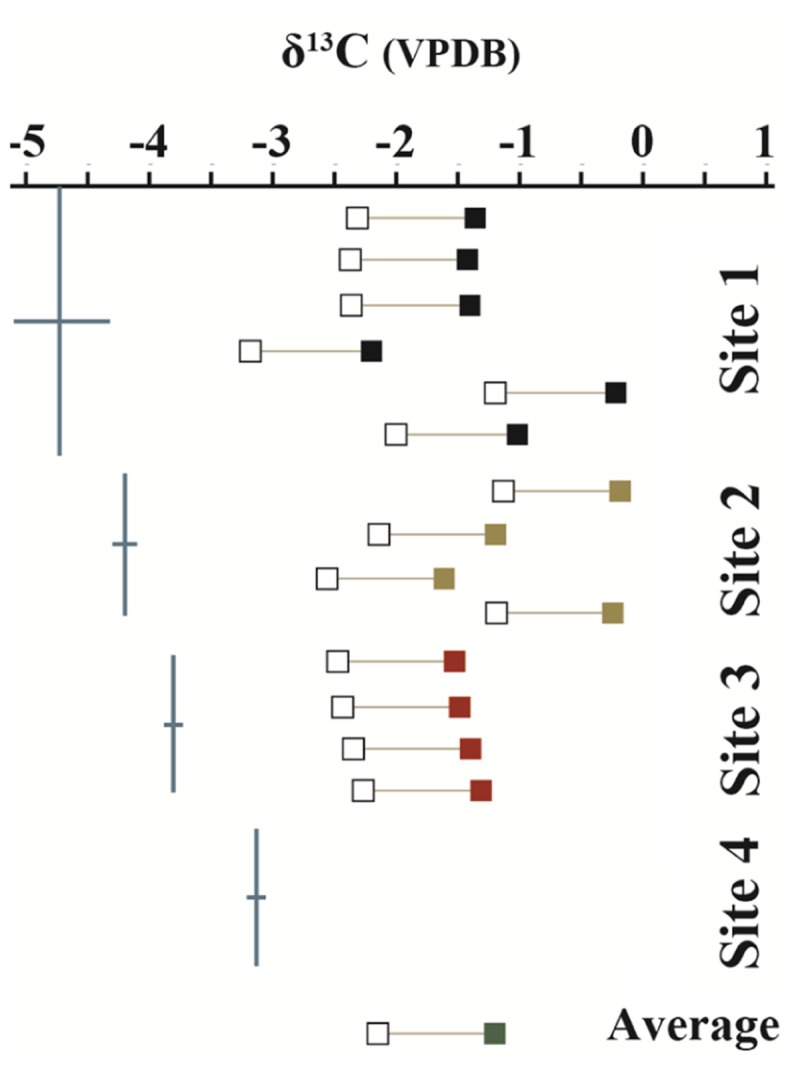
Measured δ^13^C values of mineral precipitates (closed boxes) and DIC from hot spring waters (blue lines). Open boxes show the expected isotopic values of the water from which a mineral structure precipitated under equilibrium conditions. Mineral precipitate values are plotted in order from top to bottom of sampled structure. No data was collected from the mineral precipitate of Site 4 due to the loss of the sample during transport. δ^13^C values are referenced to the Vienna PeeDee Belemnite (VPDB) standard.

The stable carbon isotopic composition of the carbonate portion of the precipitates (δ^13^C_CARB_), the measured DIC isotopic values from LHC water (δ^13^C_DIC_), and the expected isotopic values for DIC of water from which a precipitate formed (δ^13^C_EXP_), given equilibrium fractionation and a calcite-bicarbonate enrichment factor of 1.0‰ (Romanek et al., 1992), are shown in **Figure 2**. δ^13^C_CARB_ varies between -2.2 and -0.2‰ with a mean value of -1.2‰. No clear pattern is observed in δ^13^C_CARB_ from top to bottom within individual precipitates or between precipitates along the flow path. δ^13^C_EXP_ varies between -3.2‰ and -1.2‰ with a mean value of -2.2‰. δ^13^C_DIC_ is depleted relative to δ^13^C_EXP_ with values ranging from -4.8‰ and -3.1‰ and a mean value of -3.9‰. However, δ^13^C_DIC_ increases toward the average δ^13^C_EXP_ moving downstream and is comparable to the most depleted δ^13^C_EXP_ value at the final sampling location. The degassing experiment fluids exhibited a progressive decrease in DIC in association with ^13^C enrichment. The fluid DIC contents decreased from 14.7 to 13.5 mM and δ^13^C values increased from -5.2 to -3.3‰. Carbon contents and isotope composition were strongly correlated aside from the first six samples.

### Petrography

**FIGURE 3:**
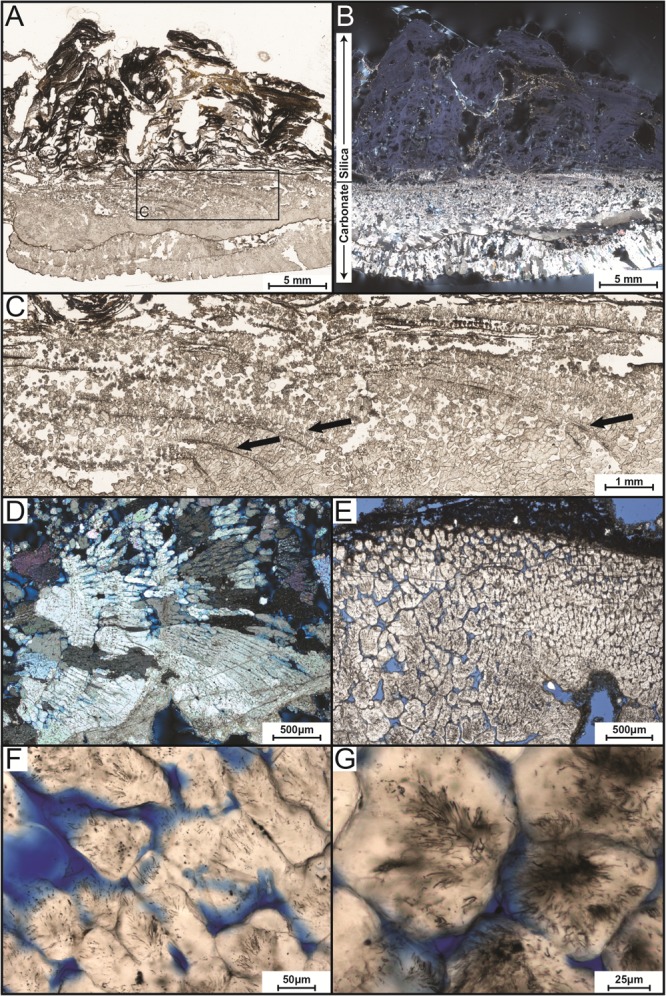
Transmitted-light petrographic photomicrographs of LHC precipitates in thin section. **(A)** Plane-polarized transmitted light photomosaic of LHC precipitate (inset box enlarged in **C**). Light-colored area toward the bottom of the image is calcite and wavy-laminated, darker area toward the top is amorphous silica. **(B)** Cross-polarized transmitted light photomosaic revealing bladed calcite crystals in lower portion and amorphous silica in the upper portion (same field of view as **A**). Amorphous silica is opaque under cross-polarized light, so a minor amount of reflected light was cast across the thin section to enhance the visibility of the upper silica portion. **(C)** Inset image from **(A)**. Arrows point to several curved, downward dipping surfaces that indicate progressive growth of the bladed calcite from left to right. **(D)** Crossed-polarized photomicrograph of bladed calcite from the lower portion of the precipitate indicating growth from left to right. **(E)** Plane-polarized transmitted light image of bladed calcite crystals with cloudy appearance caused by pervasive endolithic activity (blue areas represent pore space). **(F,G)** Plane-polarized transmitted light photomicrographs of endoliths within calcite crystals.

The precipitate sample from Site 1 near the hot spring source was used for petrographic analysis (**Figure 3A**). The precipitate was 19.5 cm long by 9 cm wide, protruded ∼2–3 cm above water surface, and extended ∼4–5 cm below the water surface. The precipitate consists of an upper, subaerially exposed, partially lithified portion and a lower, subaqueous highly lithified portion (**Figure 3B**). The top portion has a white crenulated fabric with a light-pink tinge in the center. Beneath the white domal crenulations, dark green mats were encapsulated. Along the upstream margin, the edges of the upper layer are smooth and light yellow to red in color. The lower subaqueous portion is very well lithified and black in color with a rough surface.

In thin section, two different zones were recognized, aligning with those observed in the hand sample (**Figures 3A,B**). The upper zone is laminated and consists of mixed amorphous silica and micritic calcite. The laminae are domal, wavy, and characterized by a low degree of inheritance [i.e., stromatolitic in appearance (Walter, 1976)]. The lower zone consists of bladed calcite crystal fans that grew perpendicular to or slightly downward from the surface (**Figures 3C,D**). Curved, downward dipping surfaces spaced approximately 100 μm apart are visible within the lower carbonate portion (**Figure 3C**). Within this zone, the bladed calcite crystals appear cloudy under lower magnification (**Figures 3D,E**). Higher magnification (400×) reveals a network of tubular structures (up to 2 μm in diameter and 20 μm in length) that extend into the calcite (**Figures 3F,G**).

### Microbial Communities of LHC Precipitates

**FIGURE 4:**
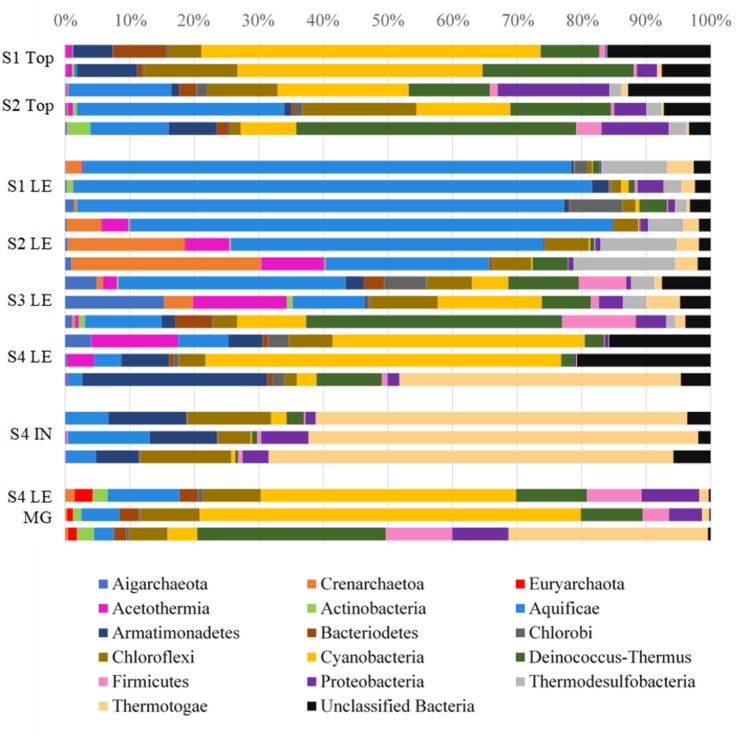
Relative abundances of bacterial and archaeal phyla in LHC precipitates. S1, S2, S3, S4 indicates Site number where a sample was taken, followed by the type of sample taken: top, leading edge (LE), precipitate interior (IN), and leading-edge metagenome (LE MG). Each bar represents the microbial community of a biological replicate. Cyanobacteria are most abundant in the precipitate top portions and at the leading edge of the Site 4 precipitate. Aquificae is heavily represented in the warmest parts of the hot spring closer to the source and Thermotogae dominates the interior mineral material.

Biofilm and lithified material were collected from leading edges and silica/calcite tops from four precipitates at four locations in LHC. SSU rRNA gene libraries were sequenced to identify microorganisms present on precipitate leading edges, tops, and interior (Accession SRX3004381). The relative abundances of bacterial and archaeal OTUs are shown at the phylum level in **Figure 4**. Eukarya were found to constitute a low percentage (0–0.7%) of the microbial community in both SSU rRNA and metagenomic data and were not investigated further for this study (Supplementary Table S1). The precipitate top communities (sampled at Sites 1 and 2) contained higher relative abundances of Cyanobacteria than the adjacent leading edges. And while no precipitate tops were sampled at Site 3 or 4, Cyanobacteria relative abundance increased at leading edges. Of the Cyanobacteria at Site 4, the metagenomic data identified the orders Chroococcales (average of 85.4% of sequences), Nostocales (6.8%), Oscillatoriales (4.6%), Prochlorales (0.8%), Gloeobacterales (1.8%), and unclassified reads (0.6%) (Supplementary Table S1). The genus *Synechococcus* comprised 90.2% of the reads attributed to Chroococcales. A maximum likelihood tree of Cyanobacteria OTUs (with greater than 100 sequences in the dataset) showed most OTUs to be most closely related to *Synechococcus* sp. JA-3-3Ab(CP000239) (Supplementary Figure S2), a photosynthetic cyanobacterium previously isolated from microbial mats in Yellowstone National Park’s Octopus Spring (Allewalt et al., 2006; Steunou et al., 2006).

Microscopy of precipitate tops indicated the presence of photosynthetic cyanobacteria populations (Supplementary Figure S1). Fluorescence microscopy revealed the most abundant cellular morphology to be rod-shaped and approximately 3 μm in length. A lesser number of coccoidal organisms was noted as well, which is the one known morphology of *Synechococcus,* the most abundant Cyanobacteria identified in sequence data. It was difficult to detect organisms entrained within the mineral grains, but when grains were examined under green light (550 nm), organisms capable of photosynthesis became more evident as they fluoresced red. Samples were stained with DAPI (4′6′-diamidino-2-phenylindole) and exposed to ultraviolet light to fluoresce DNA allowing for easier imaging of organisms associated with mineral grains. Images of mineral precipitates were taken at low magnification (200×) on an ESEM that showed filamentous organisms, potentially cyanobacteria, in close association with mineral grains on LHC precipitate tops (Supplementary Figure S1E). Under higher magnification (1200×) an amorphous matrix, potentially exopolymeric substance (EPS), appeared to be in close association with the mineral grains as well (Supplementary Figure S1F).

In addition to SSU rRNA sequencing, metagenomic libraries were generated from the leading edge of the Site 4 precipitate (Accessions mgm4709842.3, mgm4709411.3, mgm4709423.3). A total of 4,868,811 sequences were produced from 788,297,300 base pairs (Supplementary Table S2). The relative abundances of metagenomic 16S rRNA sequences associated with taxa were in general agreement with the SSU rRNA sequencing results for the sampling site (**Figure 4** and Supplementary Table S1). The metagenomic reads were run against the KEGG Orthologs database within MG-RAST to identify genes associated with photosynthesis, which could affect carbonate precipitation through the biological consumption of CO_2_ (Zhu and Dittrich, 2016). Photosynthesis and phycobilisome-associated genes were a major constituent of the metabolic makeup at Site 4, accounting for 21.6, 33.2, and 2.7% of all energy metabolism-associated sequences in samples (**Table 2**).

**Table 2:** Metabolic gene annotations from the KEGG Orthology database and MG-RAST.

	Percentage of annotated genes		
Energy metabolism	MG 4A	MG 4B	MG 4C
Oxidative phosphorylation	54.6	50.5	78.6
Photosynthesis	13.5	20.9	1.6
Photosynthesis antenna proteins	7.1	10.5	0.7
C fixation in photosynthetic organisms	1.0	1.7	0.4
C fixation in prokaryotes	5.2	3.3	8.8
Methane metabolism	10.9	6.3	6.1
Nitrogen metabolism	2.3	1.6	2.1
Sulfur metabolism	5.4	5.1	1.7

*Genes associated with photosynthetic metabolism (photosynthesis, photosynthesis antenna proteins, and carbon fixation in photosynthetic organisms) constitute 21.6, 33.2, and 2.7% of the metabolic annotations in the three samples from Site 4.*

## Discussion

### A Geochemical Model for Precipitate Formation

Metabolic reactions carried out by microorganisms have been shown to impact aqueous geochemistry and drive mineral precipitation in a number of environments (Dupraz and Visscher, 2005; Visscher and Stolz, 2005; Baumgartner et al., 2006). Thus, the intimate association of microorganisms with the studied mineral precipitates at Little Hot Creek suggests the possibility of a biological role in their formation. Indeed, photosynthesis can increase the saturation state of calcite, and genes associated with this metabolism were observed in metagenomic libraries from the Site 4 precipitate. However, the rapid physico-chemical changes that occur when the geothermal waters emerge at the surface and equilibrate with the atmosphere could also drive mineral formation. Geochemical modeling was performed to assess the effects of physico-chemical processes on the saturation states of calcite and amorphous silica, which were identified as the primary mineral phases within the precipitates at LHC from petrographic analysis. Measured geochemical values from sampling Site 1 were used as the starting parameters for all models.

**FIGURE 5:**
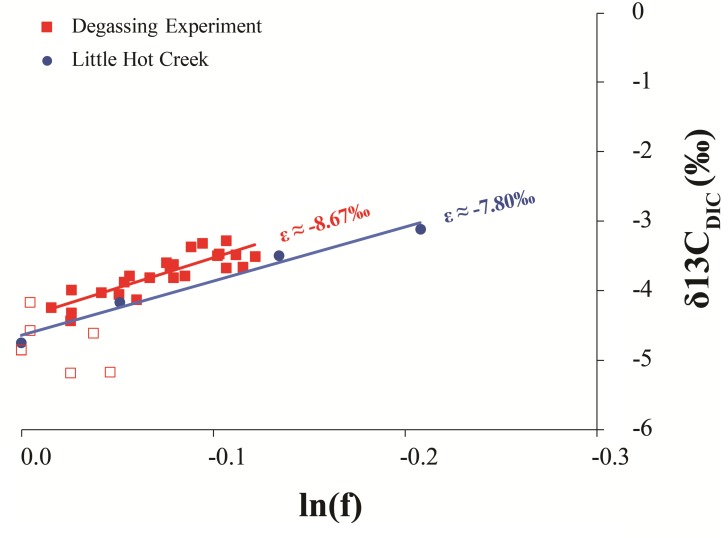
Comparison of the Rayleigh fractionation from the degassing experiment and measured values from LHC1. The slope of a regression line of the natural log of the fraction of DIC remaining in the system (lnf) and its isotopic composition (δ^13^C_DIC_) is approximately equal to the fractionation factor of the process. Open squares are the first six sampling points of the degassing experiment when mixing was likely incomplete, thus this data was not included in the regression analysis.

**Figure 5** demonstrates the progressive DIC loss and ^13^C-enrichment of LHC and source fluid experiment waters follow a Rayleigh-type evolutionary trend as has been observed in other hot springs (Usdowski et al., 1979; Michaelis et al., 1985; Fouke et al., 2000). Similar systems produce a predictable relationship between the isotope composition of the residual DIC and the fraction of DIC remaining (f). Linear regression of δ^13^C_DIC_ versus ln(f) yields a slope that is approximately equal to the fractionation (in ‰) between the lost product and reactant DIC (𝜀_P-DIC_). The fractionation associated with LHC DIC evolution (𝜀 ≈-7.80) is similar to that measured in the degassing experiment (𝜀_CO2-DIC_ ≈-8.67). Both fractionations conform to CO_2_ degassing, a common phenomenon impacting hot springs (Renaut and Jones, 2000). This suggests that other factors impacting LHC (biological and mineral precipitation related) do not yield significant isotope fractionation of spring DIC (recall that the fluid experiment was conducted outside of the spring, in the absence of spring biota and mineral precipitates). However, CO_2_ degassing can promote mineral formation by increasing pH and thus the saturation state of carbonate minerals as discussed below.

**FIGURE 6:**
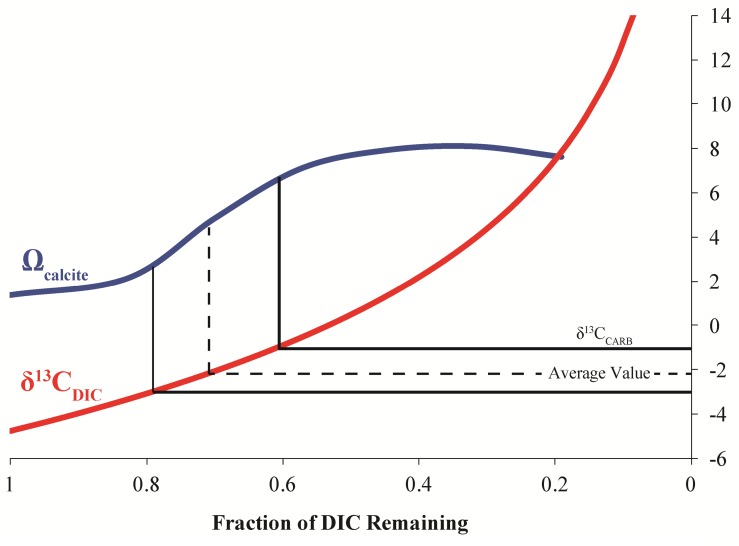
Model results for the evolution of calcite saturation state (Ω) and δ^13^C_DIC_ for degassing of LHC waters. The measured isotopic values from the precipitates (δ^13^C_CARB_) occur at the greatest slope in the modeled increase of calcite saturation state.

Because of the similarity between experimentally determined isotopic fractionations, the effect of degassing on calcite formation at LHC was modeled. Degassing was modeled by decreasing the concentration of DIC while allowing pH to vary to keep alkalinity constant as degassing removes DIC without affecting alkalinity. Model results indicate that degassing can drive an increase in the saturation state (Ω) of calcite, indicating that calcite precipitation becomes more thermodynamically favorable as CO_2_ is degassed (**Figure 6**). A Rayleigh fractionation model for carbon isotopic fractionation during degassing predicts a concurrent isotopic enrichment in carbon during the modeled rise in saturation state (Usdowski et al., 1979; Michaelis et al., 1985; Fouke et al., 2000). Intriguingly, the modeled isotopic values during the greatest increase in saturation state match those expected for waters in equilibrium with calcite measured from the precipitates (δ^13^C_EXP_). Further, the modeled saturation values are comparable to those measured at the final sampling location of LHC. The agreement between isotope compositions predicted from the model and measured values indicate that degassing alone can drive mineral formation and generate the δ^13^C values of the LHC precipitates.

**FIGURE 7:**
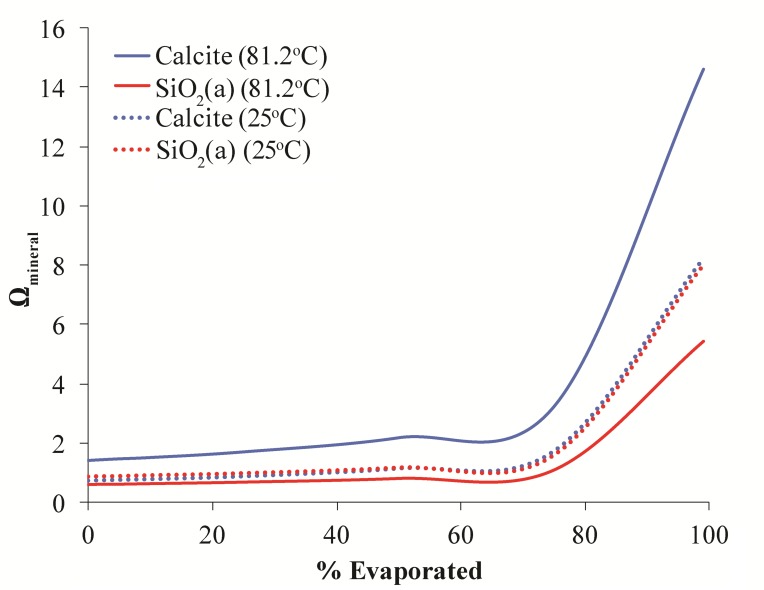
Model results for evolution of the saturation state of calcite (blue lines) and amorphous silica (red lines) during evaporation at 81.2°C and (solid lines) and 25°C (dashed lines). The saturation state of both minerals begins to increase when ∼70% of water has been evaporated. At high temperature calcite precipitation is more thermodynamically favorable than silica precipitation. However, at cooler temperatures both minerals are equally favorable.

While degassing can explain the precipitation of calcite, it has less of an effect on the saturation state of amorphous silica. Primarily, silica saturation is controlled by the concentration of dissolved silica and temperature; therefore evaporation and/or cooling of waters could potentially increase silica saturation at LHC (Berelson et al., 2011). Modeling was performed to assess the effects of these processes on silica saturation at LHC (**Figure 7**). Reducing temperature from the source temperature of 81.2–25°C alone is incapable of increasing the saturation state of silica to values where precipitation is thermodynamically favorable. Evaporation alone can drive silica to supersaturation when around 70% of water has been evaporated. However, evaporation also drives an increase of calcite saturation to values much higher than that of silica implying that calcite precipitation would be more quantitatively important in this scenario. In contrast, the combined effects of cooling and evaporation increase the saturation state of both mineral phases nearly equally, indicating that formation of each mineral would be equally favorable. This result is consistent with mixed calcite and silica layers observed petrographically and suggests that evaporation and cooling may be responsible for the precipitation of silica at LHC. Additionally, this result explains silica precipitation abiotically without any influence from microorganisms.

**FIGURE 8:**
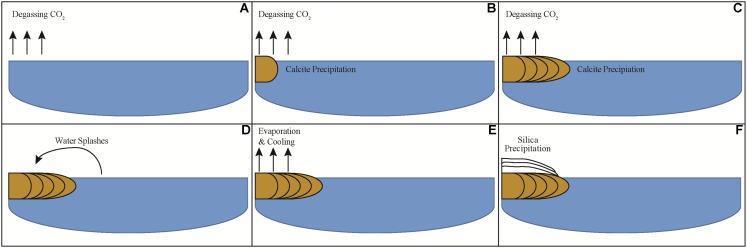
Schematic model of mineral precipitate formation at a cross-section of Little Hot Creek. **(A)** CO_2_ degasses from water increasing the saturation state for calcite. **(B)** When sufficient CO_2_ has degassed calcite begins to precipitate. **(C)** As degassing continues calcite continues to precipitate leading the mineral structure to extend perpendicularly into the stream. **(D)** When the structure extends far enough out into the stream water begins to splash onto its surface. **(E)** Water that has splashed onto the precipitate evaporates and cools. **(F)** Silica precipitates once the water cools and evaporates sufficiently.

Based on the results of geochemical modeling, a multi-step growth model was developed describing mineral precipitate formation at LHC through physico-chemical processes alone (**Figure 8**). In this model, degassing increases the saturation state of calcite moving along the flow path promoting carbonate precipitation by nucleating at a point along the edge of the stream. Sequential precipitation leads to continued calcite formation and growth of the precipitate extending into the stream, as indicated by the curved, down-dipping growth lines observed in thin section (**Figure 3C**). As the precipitate grows and extends further into the stream, it forms a platform at the air water interface where water begins to splash onto its surface, which cools and evaporates leading to the precipitation of the upper layer of amorphous silica. Water splashing onto surfaces near hot spring environments leading to silica precipitation has been observed in other hot springs (Cady and Farmer, 1996; Mountain et al., 2003). This model indicates carbonate and silica precipitation can be fully explained through degassing and evaporation alone and is consistent with the results of geochemical modeling as well as field measurements and observations. Thus, from a thermodynamic standpoint, mineral precipitate formation at LHC can be explained through wholly abiotic processes, and microbial metabolic processes are not needed to generate substantial mineral deposition in LHC although they cannot be ruled out as an additional contributor.

### Biological Controls on Precipitate Morphogenesis

The removal of CO_2_ through physio-chemical or biological processes can shift carbonate equilibrium to favor the precipitation of CaCO_3_ (e.g., Dupraz et al., 2009). Photosynthesis may drive carbonate precipitation through the biological removal of aqueous CO_2_ and the concomitant increase in alkalinity. This removal and subsequent carbon fixation process can be represented by the simplified equation: 106 CO_2_ + 16 NO_3_^-^ + HPO_4_^2-^ + 122 H_2_O + 18H^+^ → C_106_N_16_H_263_O_110_P + 138 O_2_ (Bradley et al., 2017). Metagenomic functional annotations demonstrated photosynthetic genes present in the three sampled depths at Site 4 and SSU rRNA data shows Cyanobacteria are found on precipitate tops and leading edges. Active cyanobacteria populations in LHC may be altering geochemical gradients on micrometer scales to promote localized precipitation via photosynthesis. However, LHC fluids degas CO_2_ readily as they reach the surface and equilibrate with the atmosphere along the stream’s length. Modeling results indicate carbonate and silica precipitation can be fully explained through degassing and evaporation alone, and metabolic processes are not needed to generate substantial mineral deposition. The bladed morphology of the calcite crystals is also suggestive of abiogenic precipitation (e.g., Grotzinger and Knoll, 1999; Riding, 2008), consistent with the geochemical results.

Microscale biogenic morphological features such as altered mineral structure, low inheritance between mineral layers, asymmetric laminae couples, micritization, and the inclusion of benthic microfossils can be collectively diagnostic of biotic influence (Walter and Des Marais, 1993). Inheritance and micritization are two features that can be utilized in determining biogenicity of a deposit. Inheritance describes the relationship between laminations. High inheritance, where the overlying lamination mimics the underlying lamination, indicates predominantly abiogenic precipitation. Low inheritance, where the overlying lamination differs from the underlying lamination, is typically a property of microbial mats (e.g., Grotzinger and Rothman, 1996; Grotzinger and Knoll, 1999; Frantz et al., 2014; Petryshyn et al., 2016). Micritization is the formation of micrite brought on by the alteration of minerals by endolithic microorganisms via boring and is observable as darkened granular material in petrographic thin section (Bathurst, 1966). Additionally, other trace fossils and mineral alterations can be utilized to this end, and morphological evidence of biological involvement can be highly variable across different geochemical environments. Thus, microorganisms can not only influence the precipitate morphological features during mineral formation, but also after deposition.

Petrographic and microscopic analyses suggest a microbial role in shaping the emergent silica/calcite layer of the LHC precipitates. The LHC silica/calcite layer contained domed and wavy fabrics with micritization and a low degree of inheritance that is most parsimoniously explained as the result of precipitation occurring on an irregular surface formed by a microbial mat on the precipitates. As evaporation and decreasing temperature drive precipitation of dissolved silica, it will adsorb and precipitate onto cells of microbial biofilms regardless of microbial activities (Konhauser et al., 2004; Orange et al., 2013; Spear and Corsetti, 2013). Additionally, ESEM images from this layer show microbial filaments intimately associated with minerals implying they impart some impact on mineral formation.

The presence of filamentous cells, the genomic identification of photosynthesis and Cyanobacteria on the precipitate tops and leading edges, and previous studies of hot spring microbialites indicates Cyanobacteria likely play a role in shaping the emergent precipitate, even if they did not actively play a role in mineral precipitation via metabolism or cell wall/sheath interaction. Previous studies have noted this phyla in mats on the surface of microbialites and several mm into the surface, likely due to the above layer providing protection from predation and UV light filtration through mineral grains (Walker et al., 2005). Cyanobacteria have been implicated as ‘builders’ of stromatolitic microfabrics in microbialites recovered from Obsidian Pool Prime in Yellowstone National Park, producing very finely-laminated structures with high inheritance between layers (Berelson et al., 2011; Mata et al., 2012; Pepe-Ranney et al., 2012; Spear and Corsetti, 2013), and genesis of similar microfabrics in hot spring environments due to cyanobacterial colonization have been seen elsewhere (e.g., Konhauser et al., 2001). Many of the most abundant Cyanobacteria OTUs identified in LHC are most closely related to a *Synechococcus* from Octopus Spring, another alkaline siliceous hydrothermal spring. Though environmental conditions of mineral formation between LHC and these other hot springs differ, preventing direct comparison, but instead provide insight into the variance of biogenic textures that can be produced with differing geochemistry. We postulate that cyanobacteria are involved in producing the mineral textures seen in LHC but cannot determine the mechanisms of formation. The differentiation and variability in textural clues of biogenicity highlights the need for geochemical context and environmental parameters in biosignature identification.

### Endolithic Microborings in the Abiogenic Calcite Base

In contrast to the mineral texture of the silica/calcite layer, the lower calcite layer is comprised of crystal fans that are typically interpreted as abiotic fabrics (Riding, 2008). However, the tubular dark structures observed in this abiogenic calcite layer (**Figures 3F,G**) resemble microborings made by euendoliths. Euendoliths are a type of endolithic organisms that actively bore into rock, leaving tube cavities (Golubic et al., 1981). Textural clues such as microboring distribution, size, directionality, and relation to external surfaces and fluid paths are key to determine the biogenicity of these structures (McLoughlin et al., 2007). Besides endolithic organisms, other explanations have been put forth for tunneling features in carbonates such as organic matter within rocks acting as a dissolution agent or high fluid pressures pushing harder mineral grains through a mineral precipitate (producing ambient inclusion trails) (McLoughlin et al., 2007). However, abiotic mineral dissolution at heterogeneities and ambient inclusion trails would not leave borings of the morphology we see in precipitates from LHC (see McLoughlin et al., 2010 for a review). The tube structures appear to have well-defined, circular cross-sections with similar diameters and varied lengths up to 20 μm, and the tubes propagate into the calcite from external surfaces of the crystals as if a microbe were tunneling inward.

Euendolithic cyanobacteria are commonly found in microbialites and other carbonate surfaces. It’s been suggested that these phototrophic endoliths actively transport Ca^2+^ away from the microboring front to lower the saturation state of CaCO_3_ and utilize the mineral as a carbon source in times of aqueous DIC limitation (Garcia-Pichel et al., 2010; Ramírez-Reinat and Garcia-Pichel, 2012). This mechanism allows carbonate dissolution to occur despite photosynthetic activity acting to promote carbonate precipitation and provides an explanation of the behavior as evolutionarily adaptive (Garcia-Pichel, 2006). Additionally, some lineages of Cyanobacteria (including the genus Synechococcus) are capable of generating intracellular carbonate inclusions, and this ability is not rare (Benzerara et al., 2014). Internal carbonate biomineralization could lower the alkalinity generated during photosynthesis, thereby decreasing CaCO_3_ saturation on the microscale (Benzerara et al., 2014). These metabolic interactions highlight the complexities and feedbacks in mineral-microbe relationships and how difficult it can be to tease apart such reactions.

We cannot directly demonstrate that Cyanobacteria are the euendolithic microorganisms responsible for the borings in LHC, but believe the microboring structures preserved in the calcite can act as a trace fossil of microbial activity. These endolithic signatures do not require rapid mineralization for preservation like the silica/calcite morphological biosignatures. These observations suggest that even in the absence of a direct influence of microbial activity on mineral formation, microorganisms may secondarily alter microfabrics and create biosignatures under similar environmental conditions.

## Conclusion

Examination of the biogenicity of mineral precipitates from Little Hot Creek revealed that, while abiotic processes can explain their formation thermodynamically, their morphogenesis suggests a microbial contribution, highlighting the need for interdisciplinary approaches (e.g., geochemistry, modeling, and petrography) when investigating the role of life in the formation of mineralized structures. Genomic and microscopic evidence of the microbial communities associated with the mineral precipitates indicated that Cyanobacteria were likely involved in the generation of stromatolitic microfabrics on LHC precipitate tops, while thermophiles, typical of other hot springs, reside within precipitate interiors and leading edges. This study underscores the necessity of utilizing multiple analytical tools and fields of expertise to understand the formation of geologically and astrobiologically important structures in the field of geobiology. At Little Hot Creek, geochemical investigation alone would not indicate life was involved with the formation of the precipitates. However, the addition of the microscopic textural investigation reveals a biological role in the formation and modification of the precipitates. Interpretation of potentially microbially influenced minerals from the geologic record or other planets requires a broad suite of techniques to avoid misinterpretation of the biogenicity of the structures.

## Author Contributions

JS, FC, HJ, SL, BWS, HN, and BSS led the design of the study. Fieldwork and laboratory analyses were conducted by all authors. All authors interpreted results. EK wrote the manuscript with contributions from all other authors. SB wrote the geochemical results and discussion.

## Conflict of Interest Statement

The authors declare that the research was conducted in the absence of any commercial or financial relationships that could be construed as a potential conflict of interest.

## Footnotes

1 https://omrf.org/
