# Supplementary material for: Microscale Biosignatures and Abiotic Mineral Authigenesis in Little Hot Creek, California
Source: Front Microbiol. 2018 May 25;9:997. doi: 10.3389/fmicb.2018.00997 (PMC5981138; doi:10.3389/fmicb.2018.00997)
Supplement: Supplementary file 1 [file Table_1.DOCX]

**Supplementary Information:**

**Supplementary Information - Table 1.** Taxonomic classifications of metagenomic (MG 4A – C) and SSU rRNA sequences at Kingdom level and for those sequences identified within the Phylum Cyanobacteria. SSU rRNA sequences identified most of the Cyanobacteria sequences as “Other”.

|  | **MG 4A** | **MG 4B** | **MG 4C** | **SSU rRNA, all samples** |
| --- | --- | --- | --- | --- |
| **Archaea** | 4.5 | 1.2 | 1.8 | 4.2 |
| **Bacteria** | 94.8 | 98.2 | 97.8 | 95.0 |
| **Eukaryota** | 0.7 | 0.6 | 0.5 | 0.0 |
|  | | | | 0.2 to Other* |
| **Cyanobacteria by Order** | |  |  |  |
| Chroococcales | 90.4 | 91.0 | 74.8 |  |
| Nostocales | 4.6 | 4.3 | 11.4 |  |
| Oscillatoriales | 3.4 | 3.2 | 7.3 |  |
| Prochlorales | 0.3 | 0.3 | 1.9 |  |
| Gloeobacterales | 1.0 | 0.9 | 3.5 |  |
| Unclassified | 0.3 | 0.3 | 1.1 |  |
| Total no. sequences | 189,219 | 628,813 | 21,940 |  |

**Supplementary Table 2:** Sequence library metrics for three metagenomic samples from the Site 4 precipitate. Information was taken from the MG-RAST pipeline for QC and analysis.

| **Library metric** | **MG 4A** | **MG 4B** | **MG 4C** |
| --- | --- | --- | --- |
| Sequences | 1,190,690 | 2,431,302 | 1,246,819 |
| bp | 193,326,738 | 393,009,937 | 201,960,625 |
| avg length in bp | 162 | 162 | 162 |
| % passed QC | 86.1 | 77.9 | 87.0 |
| **Of those that passed QC:** | | | |
| % ribosomal RNA | 9.0 | 11.0 | 13.0 |
| % predicted proteins w/ known function | 50.5 | 55.5 | 45.4 |
| % predicted proteins with unknown function | 40.5 | 33.3 | 42.0 |
| Post QC GC% | 56 +/- 8 | 57 +/- 8 | 54 +/- 11 |

**Supplementary Figure 1.** Light microscopy of mineral precipitate samples from LHC. (A) Brightfield image from site 3-2 indicating the primary cellular morphologies to be rod-shaped. (B) DAPI-stained microorganisms from the mineral precipitates. (C) Brightfield image of mineral grains from LHC Site 4 (D) and corresponding green light image from site 4 showing photosynthetic organisms in the mineral grains. (E) ESEM image of the Site 3 silica/carbonate portion of the mineral precipitate taken at low magnification (200x) showing potential filamentous organisms in close association with mineral grains. (F) ESEM image of the Site 4 silica/carbonate precipitate portion at 1200x. An amorphous matrix, potentially exopolymeric substance (EPS), appears in close association with the mineral grains.

**Supplementary Figure 2**. Maximum likelihood phylogenetic tree of LHC Cyanobacteria OTUs represented by greater than 100 sequences in the SSU rRNA dataset. The tree with the highest log likelihood (-3543.30) is shown. The percentage of trees in which the associated taxa clustered together is shown above the branches.
