# Supplementary figures and images for: Microscale Biosignatures and Abiotic Mineral Authigenesis in Little Hot Creek, California
Source: Front Microbiol. 2018 May 25;9:997. doi: 10.3389/fmicb.2018.00997 (PMC5981138; doi:10.3389/fmicb.2018.00997)

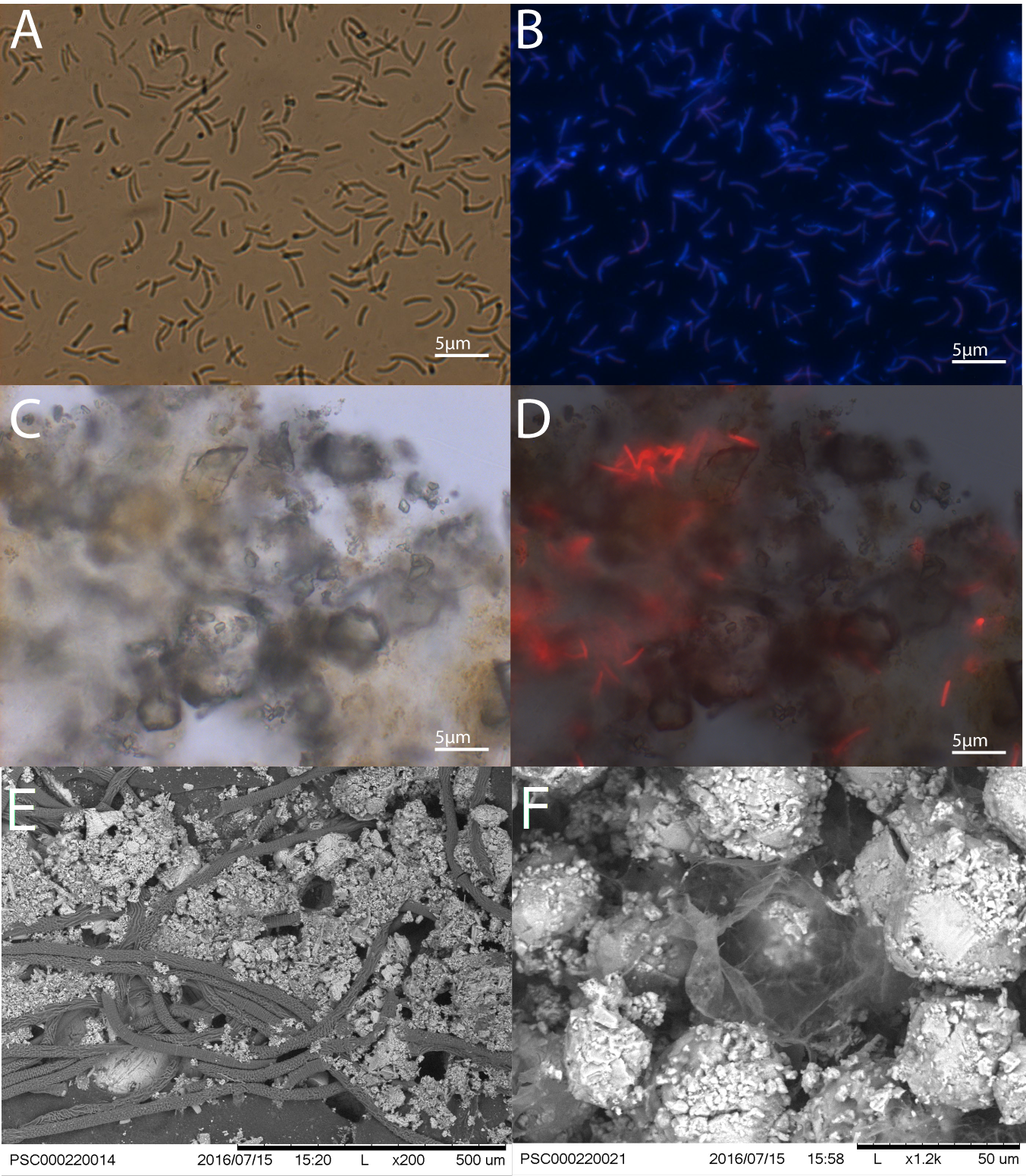

Supplement: Supplementary file 2 [file Image_1.TIF]

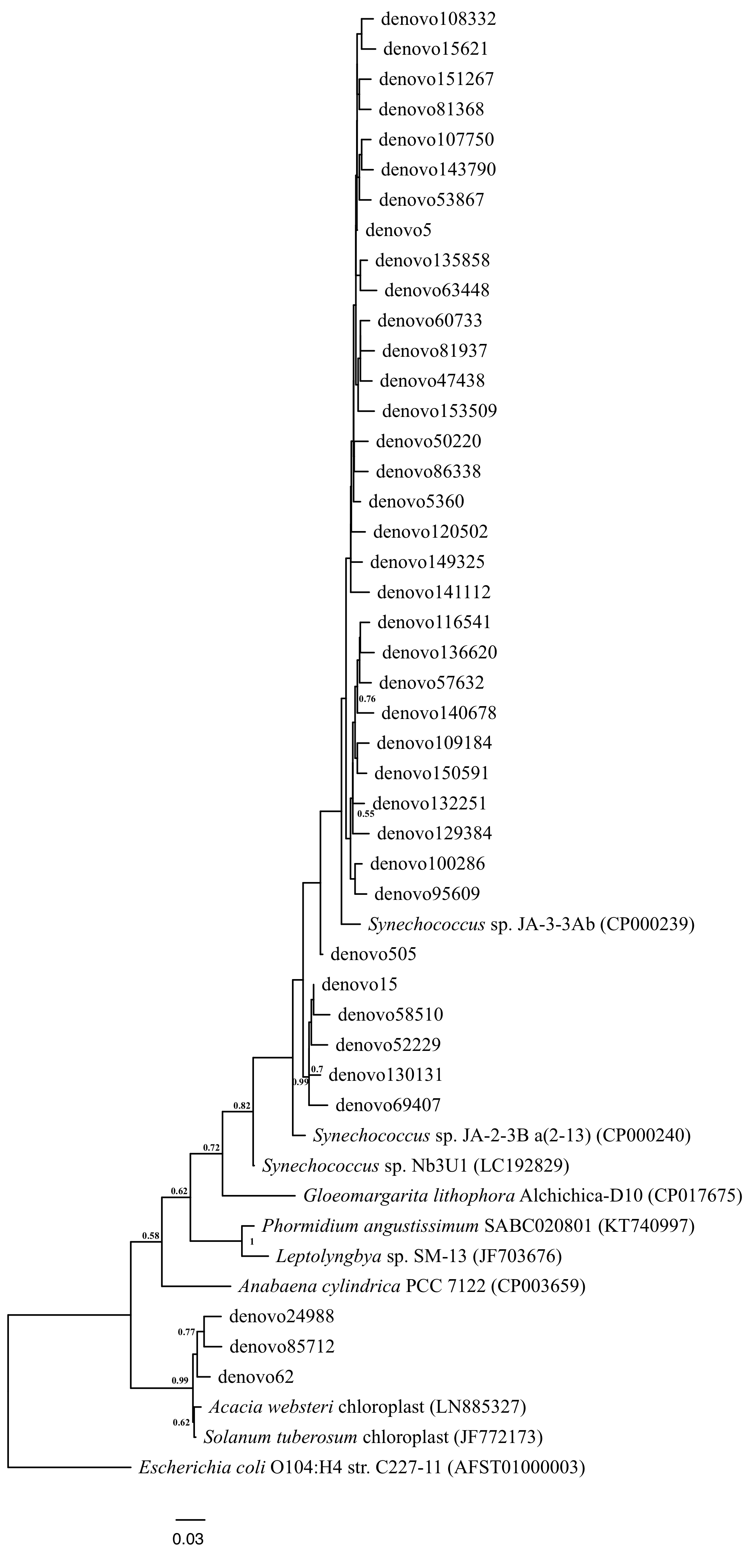

Supplement: Supplementary file 3 [file Image_2.TIF]
